# The Stability Analysis of Periodic Beams Interacting with Periodic Elastic Foundation with the Use of the Tolerance Averaging Technique

**DOI:** 10.3390/ma14205923

**Published:** 2021-10-09

**Authors:** Jakub Marczak, Jarosław Jędrysiak

**Affiliations:** Department of Structural Mechanics, Łódź University of Technology, 90-924 Łódź, Poland; jaroslaw.jedrysiak@p.lodz.pl

**Keywords:** periodic beam, elastic foundation, analytical solution, tolerance averaging technique, stability analysis

## Abstract

In this paper a stability analysis of microperiodic beams resting on the periodic inhomogeneous foundation is carried out. The main issue of this considerations, which is the analytical solution to the governing equations characterised by periodic, highly oscillating and non-continuous coefficients, is overwhelmed by the application of the tolerance averaging technique. As a result of such application, the governing equation is transformed into a form with constant coefficients which can be solved using well-known mathematical methods. In several calculation examples, the convergence of the results of two derived averaged models is examined, as well as the convergence of the lowest value of the critical force parameter derived from the averaged models with the FEM model. The results prove the superiority of the presented analytical solution over the FEM analysis in the optimisation process.

## 1. Introduction

The issue of beams resting on elastic foundations is quite common in many branches of civil and mechanical engineering. The most typical example of the application of such structures is the construction of railroads, but the concepts used in their modelling are also used to preliminarily estimate the behaviour of bridges and pipelines, etc. Due to such a wide application field, in the literature one can find many different modelling methods which are dedicated to various special engineering issues.

Since the first model of a beam resting on the elastic Winkler’s foundation was created in the middle of the nineteenth century, there was a huge step forward in the development of more sophisticated and precise models of foundations and the beams resting on them. Due to these advances, it was possible to investigate the static behaviour of more and more complex structures such as: the composite beam resting on a two-parameter elastic foundation (cf. Doeva et al. [1]), the infinite beam resting on a deformable foundation with a local subsidence (cf. Liang et al. [2]) or on tensionless foundation (cf. Zhang et al. [3]), etc. These works prove that, despite the time and computing possibilities, there are still many issues in this field which require investigations. Another branch of engineering, which is very frequently addressed in the literature is connected with the dynamic response of the considered structure to the external loading. Such a case was investigated by Javadi and Rahmanian [4], who examined the nonlinear vibrations of a fractional Kelvin–Voigt viscoelastic beam; by Abdoos et al. [5], who investigated the response of curved beams to a moving mass; and by Hien et al. [6], who used a spring-damper-mass system to model a random vehicle moving through the beam. Similar works may lead to the creation of a specific theoretical framework, which could have an outstanding practical application, such as in the damage detection of railway tracks, proposed by Yang et al. [7]; hence, this topic is well worth studying.

In this work the issue of the buckling analysis of a beam resting on the elastic foundation is investigated. Such an issue is widely covered in the literature in many special engineering cases such as: the stability of an asymmetric sandwich beam subjected to a pulsating axial load (cf. Pradhan and Dash [8]), the stability of a functionally graded sandwich beam (cf. Tossapanon and Wattanasakulpong [9]), the buckling analysis of a double-functionally graded Timoshenko beam system (cf. Deng et al. [10]), the buckling of thin-walled, functionally graded sandwich I-beams (cf. Nguyen et al. [11]) or the stability of a Rayleigh beam under moving loads (cf. Kim [12]). Unfortunately, all of the mentioned works consider only beams with either constant or functionally graded cross-sections. Moreover, the foundation parameters should also be constant for the whole structure, which creates many limitations, especially during the optimisation process. 

In order to overcome these limitations, the method of the analysis of a microperiodic beam interacting with a periodic heterogeneous foundation is proposed in this paper. The main issue with this method is that all the material properties and parameters describing the geometry of the considered structure can be provided by the periodic, highly oscillating and non-continuous functions and, as a consequence, the governing equations describing its behaviour are also provided by partial differential equations with non-constant coefficients. The solution to these equations can be obtained using either a homogenisation method, which is not capable of taking into account the effect of the microstructure size on the overall behaviour of the beam, or a numerical approach, which can be a time-consuming process which requires many computing resources. This is why, in this work, the derived governing equations are transformed by the tolerance averaging technique into a form with constant coefficients, which afterwards can be easily solved using well-known mathematical methods. The proposed algorithm of the calculations based on the tolerance averaging technique is widely used in many different mechanical issues, such as: the stability of visco-elastic beams (cf. Jędrysiak [13,14]), stability of cylindrical shells (cf. Tomczyk and Szczerba [15], Tomczyk et al. [16]), dynamics of beams (cf. Domagalski [17], Domagalski et al. [18], Domagalski and Jędrysiak [19]), statics of plates with a dense system of ribs (cf. Marczak et al. [20]), dynamics of sandwich plates (cf. Marczak [21]) and various thermomechanical issues as well (cf. Kamiński and Ostrowski [22], Ostrowski and Jędrysiak [23], Kubacka and Ostrowski [24], Pazera and Jędrysiak [25], Ostrowski and Michalak [26]).

## 2. Modelling Foundations

**Figure 1:**
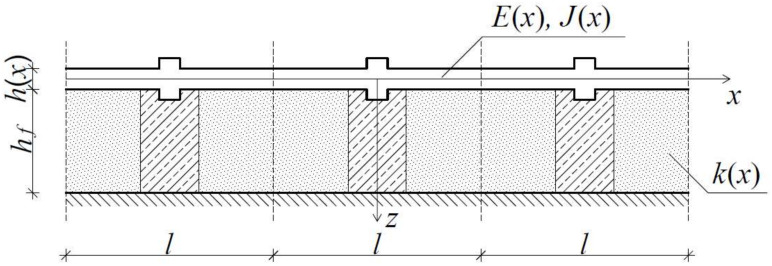
Beam with a certain periodic microstructure resting on the non-uniform foundation.

Let us denote *0x_1_x_2_x_3_* as an orthogonal Cartesian coordinate system, where $x\equiv x_{1}$ and $z\equiv x_{3}$. The considered beam is assumed to have a span *L* along the *x*-axis direction, a thickness *h*(*x*) along the *z*-axis direction, and a constant width *b* along *x*_2_-axis direction. In all our considerations it is assumed that the whole structure is made from isotropic materials described by Young’s modulus *E*(*x*) and that it interacts with a Winkler’s type foundation, which is described by the parameter *k*(*x*), cf. Figure 1.

As it can be already observed, several of the introduced parameters, such as: Young’s modulus *E*(*x*), the thickness of the beam *h*(*x*) and the modulus of the foundation *k*(*x*), are functions of the *x*-coordinate. This is caused by the fact that both the considered beam and its foundation can be characterised by a certain periodic microstructure. By analysing the mentioned microstructure, it is possible to distinguish a small, repeatable element, called the periodicity cell Δ. In our case, let us assume, that the periodicity cell has the dimension *l* along the *x*-axis direction, which is referred to as microstructure parameter. Eventually, for the sake of simplicity, we denote a spatial derivative as: $\partial \equiv \frac{\partial }{\partial x}$.

The initial point of our modelling procedure is the formulation of the displacement hypothesis. Let us assume that the considered beam fulfils all the conditions of the well-known Bernoulli’s beam theory. Moreover, by assuming the stress–strain relation according to Hooke’s law, it is possible to derive the initial governing equation of the considered structure in the following form:

(1) $$\partial \partial \left[E(x)J(x)\partial \partial w(x)\right]-\partial \left[n\partial w(x)\right]+k(x)w(x)=q(x)$$

where: *J*(*x*) is the second moment of the area of the cross-section, *w*(*x*) is the displacement of the midplane of the beam along the *z*-axis direction, *n* is an axial force, constant on the whole structure and *q*(*x*) is the set of external loadings acting perpendicularly to the beam’s axis. Notably, the presented Equation (1) is the most basic equation of the beam interacting with a Winkler’s type foundation. What is unusual about this equation is that its coefficients can be provided as periodic, highly oscillating and non-continuous functions of the *x* coordinate, which makes it very difficult to solve. In the next step of modelling, the Equation (1) is transformed into a form with constant coefficients with the use of the tolerance averaging technique.

## 3. Tolerance Averaging Technique

The modelling procedure, which leads to the derivation of the governing equations with constant coefficients, is based on the *tolerance averaging technique*. The precise description of all concepts of this technique can be found in the literature, cf. Woźniak et al. [27,28]. In this section only a physical sense of several basic concepts is presented.

Let us start with a definition of the *tolerance parameter* δ, which is an arbitrary positive number. In the whole modelling process it is assumed that certain terms, with a difference smaller than the tolerance parameter δ, can be treated as equals. In addition, by analysing the close surrounding of a basic periodicity cell Δ of this structure, it is possible to define different types of functions such as:

- *tolerance periodic function*, $TP_{\delta }^{k}(\Delta )$, which is a periodic function on the considered region with respect to tolerance parameter δ;
- *slowly varying function*, $SV_{\delta }^{k}(\Delta )$, which is a constant function on the considered region with respect to tolerance parameter δ;
- *fluctuation shape function*, $FS_{\delta }^{k}(\Delta )$, which represents the fluctuations of a certain physical field caused by the periodic microstructure of the considered structure.

Eventually, one should mention the definition of an averaging operator, which for a 1D issue can be presented in the form:

(2) $$<\partial^{k}f>(x)=\frac{1}{l}\int_{\Delta (x)}{\tilde{f}}^{\left(k\right)}(x,y)dy,\;k=0,1,2,\ldots ,$$

where ${\tilde{f}}^{\left(k\right)}$ is a periodic approximation of the *k*^th^ gradient of function f.

There are two main assumptions of the tolerance averaging technique. The first of them is the micro-macro decomposition, according to which a specific physical field *w*(·) can be expressed as a sum of the averaged macrofield of that physical property *W*(·) and a product of the fluctuation shape functions *h^A^*(·) and their amplitudes *V^A^*(·):

(3) $$\begin{array}{l} w(\cdot )=W(\cdot )+h^{A}(\cdot )V^{A}(\cdot ),\\ W(\cdot ),V^{A}(\cdot )\in SV_{\delta }^{2}(\Delta ),\;h^{A}(\cdot )\in FS_{\delta }^{2}(\Delta ),\;A=1,2,\ldots ,M. \end{array}$$

Both macrofield *W*(·) and fluctuation amplitudes *V^A^*(·) are assumed to be slowly varying functions, which means, that they can be treated as constants on the basic periodicity cell Δ.

The second assumption is a set of tolerance averaging approximations from which the averaged terms can be simplified into the most convenient form. By introducing the provided a priori tolerance parameter δ, it is possible to prove the relations:

(4) $$\begin{array}{ll} <\phi >(\cdot )=<\tilde{\phi }>(\cdot )+O(\delta ), & <\phi F>(\cdot )=<\phi >(\cdot )F(\cdot )+O(\delta ),\\ <\phi \partial \left(gF\right)>(\cdot )=<\phi \partial g>(\cdot )F(\cdot )+O(\delta ),\\ <g\partial (\phi \Phi )>(\cdot )=-<\phi \Phi \partial g>(\cdot )+O(\delta ), & 0<\delta <<1,\\ \phi ,\Phi \in TP_{\delta }^{2}(\Delta ),\;F\in SV_{\delta }^{2}(\Delta ), & g\in FS_{\delta }^{2}(\Delta ), \end{array}$$

where $\tilde{\phi }$ is periodic approximation of function ϕ and O(δ) is a negligibly small term.

## 4. The Equations of the Averaged Models

Within the tolerance averaging technique there are several different modelling approaches. Some of them are used to average the equations obtained with the use of variational methods, while others are based on the orthogonalisation condition. All of those approaches are described in detail in the works of Woźniak [27,28]. In this paper two different modelling procedures which are based on the orthogonalisation condition, are presented and discussed.

### 4.1. Tolerance Model

In order to derive a tolerance model of the considered structure, several steps of the modelling must be performed. Firstly, the whole structure must be divided into a set of small repeatable elements, called *periodicity cells*. Secondly, the initial governing Equation (1) is formulated for such a distinguished periodicity cell and a form of micro-macro decomposition (3) of the displacement field is assumed and introduced into this equation. In the next step the whole equation is averaged with the averaging operator (2) and a set of orthogonalisation conditions for the obtained averaged equation and arbitrarily chosen fluctuation shape functions are formulated. Eventually, the use of the tolerance averaging approximations (4) is required in order to obtain the most convenient form of equations.

As a result of the described modelling procedure, where the micro-macro decomposition of the displacement field can be formulated as:

$$\begin{array}{ll} w(x)=W(x)+h^{A}(x)V^{A}(x),\\ W(x),V^{A}(x)\in SV_{\delta }^{2}(\Delta ),\;h^{A}(x)\in FS_{\delta }^{2}(\Delta ), & \;A=1,2,\ldots ,M, \end{array}$$

where W(x) is the macrodisplacement function, $V^{A}(x),A=1,2,\ldots ,M,$ are the fluctuation amplitude’s functions and $h^{A}(x),A=1,2,\ldots ,M,$ are the fluctuation shape functions, one can obtain a set of governing equations for the tolerance model (TM) of the periodic beam resting on the periodic foundation in the following form:

(5) $$\begin{array}{l} D\partial \partial \partial \partial W+D^{A}\partial \partial V^{A}+KW+\underline{l^{2}K^{A}V^{A}}-N\partial \partial W-Q=0,\\ (D^{AB}+\underline{l^{4}K^{AB}})V^{B}+D^{A}\partial \partial W-\underline{l^{2}H^{AB}NV^{B}}+\underline{l^{2}K^{A}W}-\underline{l^{2}Q^{A}}=0, \end{array}$$

where:

(6) $$\begin{array}{lll} D\equiv <EJ>, & D^{A}\equiv <EJ\partial \partial h^{A}>,\; & D^{AB}\equiv <EJ\partial \partial h^{B}\partial \partial h^{A}>,\\ K\equiv <k>, & l^{2}K^{A}\equiv <kh^{A}>, & l^{4}K^{AB}\equiv <kh^{A}h^{B}>,\\ l^{2}H^{AB}\equiv <\partial \partial h^{A}h^{B}>,\; & N\equiv <n>,\\ Q\equiv <q>, & l^{2}Q^{A}\equiv <qh^{A}>. \end{array}$$

Depending on the amount of assumed fluctuation shape functions $h^{A}(x),A=1,2,\ldots ,M,$ we arrive at the system of *M+*1 partial differential equations with constant coefficients. In order to solve the above system of equations one should formulate four boundary conditions for the macrodeflection function *W*(*x*). Let us notice that there is no need for the formulation of any boundary condition for the fluctuation amplitudes *V^A^*(*x*). The underlined terms depend on the microstructure parameter *l*.

### 4.2. Asymptotic Model

The procedure of deriving the asymptotic model of the periodic beam resting on the periodic foundation is similar to the procedure presented in Section 4.1. Firstly, the whole structure must be divided into a set of small repeatable elements, called *scaled periodicity cells* $\Delta_{\varepsilon }$ with the parameter ε. Secondly, the initial governing Equation (1) is formulated for such a distinguished, scaled periodicity cell and a form of asymptotic decomposition of the displacement field is assumed and introduced into this equation. The general form of asymptotic decomposition of any physical field can be expressed with:

$$\begin{array}{lll} w(\cdot ,y)=W(y)+\varepsilon^{2}h_{\varepsilon }{}^{A}(\cdot ,y)V^{A}(y),\; & A=1,\ldots ,M;\;\\ h_{\varepsilon }{}^{A}(\cdot ,y)=h^{A}(\cdot ,\frac{y}{\varepsilon }),\;\varepsilon =1/p, & p=1,2\ldots , & y\in \Delta_{\varepsilon }(\cdot ); \end{array}$$

where W(y) is the macro displacement function, $V^{A}(y),A=1,2,\ldots ,M,$ are the fluctuation amplitude’s functions and $h^{A}(x,y),A=1,2,\ldots ,M,$ are the fluctuation shape functions. One can then obtain a set of governing equations of the asymptotic tolerance model (ATM) of the periodic beam resting on the periodic foundation in the following form:

(7) $$\begin{array}{l} D\partial \partial \partial \partial W+D^{A}\partial \partial V^{A}+KW-N\partial \partial W-Q=0,\\ D^{AB}V^{B}+D^{A}\partial \partial W=0, \end{array}$$

where all the denotations from Section 4.1 apply.

Depending on the amount of assumed fluctuation shape functions $h^{A}(x,y),A=1,2,\ldots ,M,$ we arrive at the system of *M+*1 partial differential equations with constant coefficients. However, this system of equations can be transformed into one differential equation with only one unknown function *W*(*x*). In order to solve the above equation, one should formulate four boundary conditions for the macrodeflection function *W*(*x*). It can be noticed that exactly the same system of Equation (7) can be derived from Equation (5) by neglecting the terms which are dependent on the microstructure parameter *l*.

## 5. Calculation Examples

In this section several different calculation cases are presented and discussed. Firstly, in the case with only one fluctuation shape function, the general formulas for the critical force are derived from the governing equations of TM and ATM. These formulas are then used to compare the results of the two presented averaged models in a large-scale buckling analysis of certain periodic structures. Eventually, for another set of periodic structures, the lowest values of the critical force obtained within the two models are compared with the results obtained within the FEM model. The aim of this analysis is the determination of parameters, which can cause significant discrepancies in results, while proving both the correctness and superiority of the presented analytical models over the FEM model.

### 5.1. Derivation of Formulas for Critical Force Parameters

**Figure 2:**
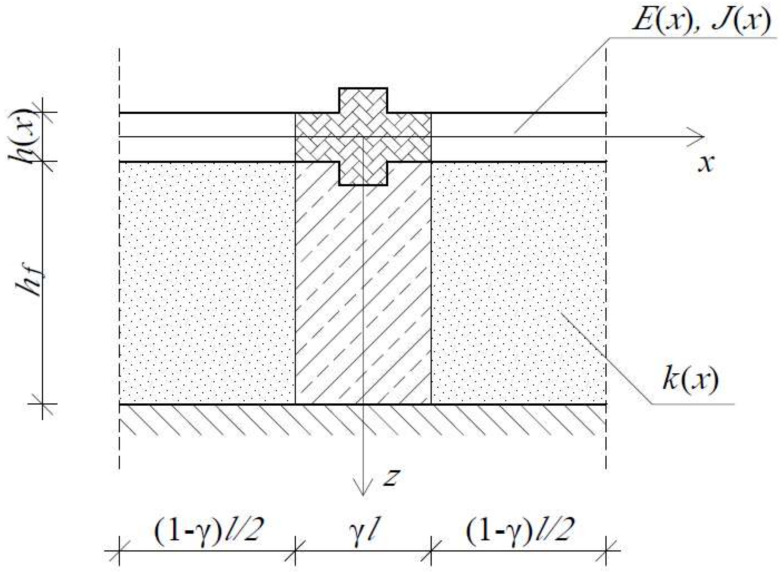
A considered basic periodicity cell of the beam resting on a non-uniform foundation.

Let us consider a simply supported microperiodic beam resting on the periodic elastic foundation. The beam fulfils all the modelling conditions which are presented in Section 2. Moreover, its periodicity cell can be defined as presented in Figure 2, where γ is a dimensionless parameter γ∈<0,1>.

Let us analyse the system of the governing equations of TM (5). By assuming only one fluctuation shape function $h^{1}(x)$, the presented system of governing equations is limited to only two equations:

(8) $$\begin{array}{l} D\partial \partial \partial \partial W+D^{1}\partial \partial V^{1}+KW+\underline{l^{2}K^{1}V^{1}}-N\partial \partial W-Q=0,\\ (D^{11}+\underline{l^{4}K^{11}})V^{1}+D^{1}\partial \partial W-\underline{l^{2}H^{11}NV^{1}}+\underline{l^{2}K^{1}W}-\underline{l^{2}Q^{1}}=0, \end{array}$$

where all the denotations (6) apply. Assuming that the solutions to the system of Equations (8) are in the form which satisfies the simply supported boundary conditions:

(9) $$\begin{array}{cc} W(x)=A_{W}\sin(\lambda x),\; & V\equiv V^{1}(x)=A_{V}\sin(\lambda x), \end{array}$$

where $A_{W},A_{V}$ are amplitudes of macrodisplacements and fluctuations, respectively, and λ is a wave number λ=mπ/L,m=1,2,…, the critical force parameters can be evaluated using the following formulas:

(10) $$\begin{array}{ll} {\tilde{F}}_{-}\equiv & \frac{H^{11}l^{2}\left(\lambda^{2}D+\lambda^{-2}K\right)-\left(D^{11}+l^{4}K^{11}\right)}{2H^{11}l^{2}}+\\ & \;-\frac{\sqrt{{\left[H^{11}l^{2}\left(\lambda^{2}D+\lambda^{-2}K\right)+\left(D^{11}+l^{4}K^{11}\right)\right]}^{2}-4H^{11}l^{2}{\left(\lambda D^{1}-l^{2}\lambda^{-1}K^{1}\right)}^{2}}}{2H^{11}l^{2}},\\ {\tilde{F}}_{+}\equiv & \frac{H^{11}l^{2}\left(\lambda^{2}D+\lambda^{-2}K\right)-\left(D^{11}+l^{4}K^{11}\right)}{2H^{11}l^{2}}+\\ & \;+\frac{\sqrt{{\left[H^{11}l^{2}\left(\lambda^{2}D+\lambda^{-2}K\right)+\left(D^{11}+l^{4}K^{11}\right)\right]}^{2}-4H^{11}l^{2}{\left(\lambda D^{1}-l^{2}\lambda^{-1}K^{1}\right)}^{2}}}{2H^{11}l^{2}}. \end{array}$$

It should be emphasised that the presented formulas for the critical force parameter always take exactly the same form regardless of the type of inhomogeneity present in the periodicity cell. Hence, exactly the same formulas can be used to evaluate a critical force parameter for the homogeneous beam resting on the periodic foundation, the periodic beam resting on the homogeneous foundation and the periodic beam resting on the periodic foundation (the form of the microstructure is taken into account by coefficients (6), evaluated for each calculation case). The versatility of the presented model is one of its greatest advantages.

Let us now derive a similar formula from the system of Equations (7). Taking into account exactly the same assumptions as the previous derivations, the system of governing equations can be written as follows:

(11) $$\begin{array}{l} D\partial \partial \partial \partial W+D^{1}\partial \partial V^{1}+KW-N\partial \partial W-Q=0,\\ D^{11}V^{1}+D^{1}\partial \partial W=0. \end{array}$$

The solutions to the system of Equations (11) can be assumed in the form of (9), which satisfies the simply supported boundary conditions. Eventually, we arrive at the critical force parameter formula:

(12) $$\tilde{F}=\{[D-D^{1}{\left(D^{11}\right)}^{-1}D^{1}]\lambda^{4}+K\}\lambda^{-2},$$

which can also be used in the buckling analysis of any type of periodic, inhomogeneous beam resting on the periodic foundation. Both Formulas (10) and (12) are used in the subsequent calculation examples.

### 5.2. Example of Calculations I—The Analysis of Discrepancies between the Averaged Models

In this section the discrepancies between the two presented averaged models are analysed in the large-scale buckling analysis of several microperiodic beams. Let us consider a simply supported beam, which basic periodicity cell is presented on Figure 2. For such a structure, let us introduce several relations between the dimensions of the structure and its parameters describing material properties:

$$\begin{array}{l} b=0.1l,\;L=30l,\;\lambda =\frac{m\pi }{L},\;m=0,1,\ldots \\ h(x)=\left\{\begin{array}{lll} 0.07l & for & x\in <-l/2,-l/10)\\ 0.1l & for & x\in <-l/10,l/10>\\ 0.07l & for & x\in (l/10,l/2> \end{array}\right.,\\ E(x)=E,\\ k(x)=\left\{\begin{array}{lll} \xi E & for & x\in <-l/2,-l/10)\\ 0.05E & for & x\in <-l/10,l/10>\\ \xi E & for & x\in (l/10,l/2> \end{array}\right., \end{array}$$

where ξ is a dimensionless parameter. By assuming the only fluctuation shape function in the form of an even function:

(13) $$h^{1}(x)=l^{2}\cos\left(2\pi x/l\right),$$

it is possible to evaluate the critical force parameters according to Formulas (10) and (12). All calculations are performed for three cases, which differ from each other with parameter ξ:

- Case I—ξ=0.015;
- Case II—ξ=0.02;
- Case III—ξ=0.025.

For the sake of simplicity, all the results are presented in the dimensionless form obtained by the following transformations:

$$\begin{array}{lll} F_{TM-}={\tilde{F}}_{-}/El^{2}, & F_{TM+}={\tilde{F}}_{+}/El^{2}, & F_{ATM}=\tilde{F}/El^{2},\\ R_{-}=F_{TM-}/F_{ATM},\; & R_{+}=F_{TM+}/F_{ATM}.\; \end{array}$$

**Figure 3:**
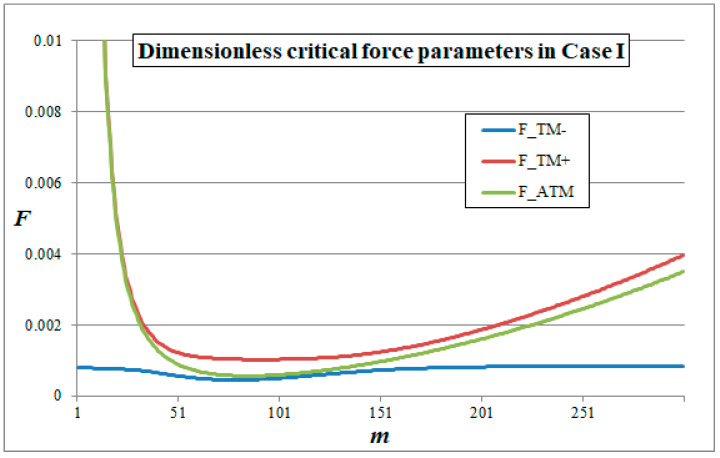
Dimensionless critical force parameters versus wave number *m* according to averaged models in Case I.

**Figure 4:**
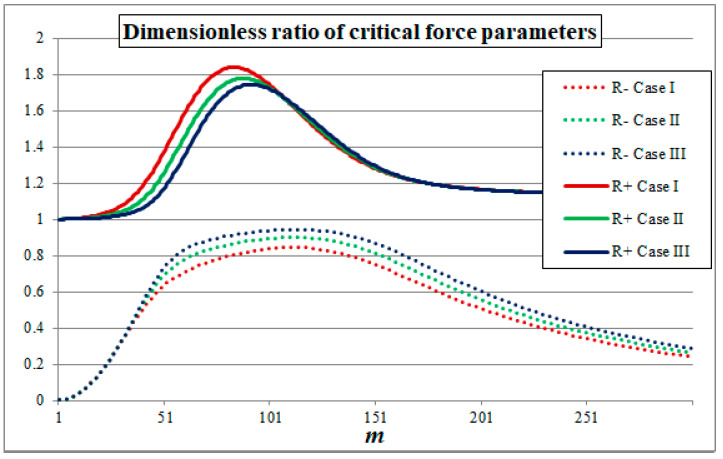
Dimensionless ratio between critical force parameters of TM to ATM.

The results of the comparisons are presented in Figure 3, which shows the dimensionless critical force parameters obtained within TM and ATM in Case I for a wide range of wave numbers *m*. Similar diagrams can be obtained for other cases. In order to present a general trend, the results of all the analysed cases are gathered in Figure 4, where the dimensionless ratios of the critical force parameters are presented.

By analysing Figure 3 and Figure 4 one can observe a sufficient convergence of the results of TM and ATM in a wide scope of wave numbers. Due to a specific form of governing equations, within the TM it is possible to obtain two different values of critical force parameters for each wave number *m*. The lower value $F_{TM-}$ represents the macroscale buckling modes, while the higher value $F_{TM+}$ represents the microscale buckling modes, which are the result of the periodic microstructure. It can be noticed that, depending on the value of *m*, the results of either $F_{TM-}$ or $F_{TM+}$ can be considered convergent with $F_{ATM}$ or at least can represent the same tendency as $F_{ATM}$. The differences in those results are due to the effect of the simplifications made in ATM, such as the limit passage with the dimension of the periodicity cell to zero. Nevertheless, from an engineering point of view the most important factor is the lowest value of the critical force parameter, which is investigated in the subsequent subsection.

### 5.3. Example of Calculations II—The Lowest Critical Force Parameter

In these examples of calculations let us focus on the evaluation of the first, lowest value of the critical force, which is the most significant parameter for the engineers. This analysis is performed for two sets of simply supported microheterogeneous beams, for which the periodicity cell can be presented as in Figure 2. Let us define those two sets:

- Set I—the isotropic periodic beam with a constant thickness resting on the uniform foundation:

$$\begin{array}{l} b=3\mathrm{cm},\;h(x)\equiv h=2.1\mathrm{cm},\;L=6m,\\ E=210\mathrm{GPa},\;k(x)=k,\\ E(x)=\left\{\begin{array}{lll} \xi E & for & x\in <-l/2,-\gamma l/2)\\ E & for & x\in <-\gamma l/2,\gamma l/2>\\ \xi E & for & x\in (\gamma l/2,l/2> \end{array}\right.. \end{array}$$

- Set II—the isotropic homogeneous beam with a constant thickness resting on the periodic foundation:

$$\begin{array}{l} b=3\mathrm{cm},\;h(x)\equiv h=2.1\mathrm{cm},\;L=6m,\\ E=210\mathrm{GPa},\;k_{1}=100\mathrm{kPa},\\ k(x)=\left\{\begin{array}{lll} k_{2} & for & x\in <-l/2,-\gamma l/2)\\ k_{1} & for & x\in <-\gamma l/2,\gamma l/2>\\ k_{2} & for & x\in (\gamma l/2,l/2> \end{array}\right.. \end{array}$$

The aim of such a distinction is the indication of the parameters, which can cause some discrepancies in the results. Both sets of beams are analysed with the use of both averaged models (TM and ATM) in order to find the lowest value of the critical force. The modelling procedure requires the definition of the fluctuation shape function, which in this example of calculations is set as in Formula (13).

Eventually, the obtained results are compared with the results of the FEM models prepared and evaluated in the Abaqus environment. The beams were modelled with 2D shell elements with proper boundary conditions and interactions with elastic foundations. The obtained results are presented in Table 1, Table 2, Table 3, Table 4, Table 5, Table 6, Table 7, Table 8. For the sake of conciseness, the results are presented only in the form of relative errors between the averaged models and the FEM models.

By analysing Table 1, Table 2, Table 3, Table 4, Table 5 and Table 6, one can conclude that the lowest critical force parameter obtained within both the TM and ATM is convergent with the FEM analysis in a wide range of calculation cases, including various material distribution and its properties within the periodicity cell. In general, it can be stated that the homogeneous foundation differences between the averaged models and the FEM models are negligibly small when the periodicity cell is made of materials, with an elastic modulus ratio ξ∈<0.5,1>. For such structures the relative errors between the analytical and numerical approaches usually do not exceed 2% and they are only slightly affected by the distribution of materials in the periodicity cell. Such results prove the correctness of the tolerance modelling procedure. For structures made with more diverse materials these relative errors may be higher and reach up to 10–30% in extreme cases, where ξ<0.2. Due to such discrepancies, the modelling of such structures should not be performed with the averaged models at all.

The results presented in Table 7 and Table 8 concern the homogeneous beams resting on the periodic foundations. In such cases the outstanding convergence of results between both averaged models and the FEM analysis can be observed regardless of the type of inhomogeneity. The presented results prove that, even in the case of huge discrepancies between the foundation parameters, the derived averaged models can be used to estimate the first critical force parameter.

## 6. Discussion and Conclusions

In this paper the stability analysis of the microperiodic beams resting on the periodic inhomogeneous foundation is performed. The main issue of this method, which is the analytical solution to the governing equations characterised by periodic, highly oscillating and non-continuous coefficients, is overcome by the application of the tolerance averaging technique. As a result of this technique, the governing equation is transformed into a form with constant coefficients which can be solved using well-known mathematical methods.

Within this paper, two different tolerance modelling procedures are used to obtain two averaged models of the considered structure: TM and ATM. Within the calculation examples, the formulae for the critical force parameters for a simply supported beam with any kind of inhomogeneity are derived and used to investigate the accuracy of the presented solution. Even though the convergence of the proposed averaged solutions can be considered questionable in a large-scale buckling analysis (cf. Section 5.2), it should be emphasised that both models are exceptionally convergent when it comes to the evaluation of the first, lowest critical force parameter, which is crucial from a practical point of view. In such a case, the use of ATM is recommended, due to a significant simplicity of Formula (12) in comparison to Formula (10).

In Section 5.3 two sets of periodic structures are analysed in order to distinguish a parameter which can cause some discrepancies in the results between the averaged models and the FEM model. Based on the presented analysis, the specific modelling limitations of the presented solutions can be formulated as follows:

- The precision of the averaged models is exceptional for structures, with an elastic modulus ratio ξ∈<0.5,1>. For structures which do not fulfil this condition the results of the averaged models can be affected by a considerable relative error;
- The influence of the inhomogeneous foundation on the precision of the averaged models is negligibly small.

These two points should always be taken into consideration before the estimation of the lowest critical force parameter. Let us notice that in all the considered calculation cases the modulus of the foundation *k*(*x*) is assumed to be significantly lower than the modulus of elasticity of the beam *E*(*x*). The issue, in which those two parameters are of the same order, may require the formulation of additional or different rules of applicability. In the final analysis, the formulation of the exact model of the periodic beam resting on the periodic foundation may also be necessary to estimate the precision of the averaged models.

The greatest finding of the proposed averaged models is that they are capable of determining the behaviour of microheterogeneous structures with the use of the partial differential equations with constant coefficients. Moreover, the type of inhomogeneity has no influence on the final form of the governing equations. As a result, exactly the same model can be used to analyse a wide variety of periodic structures, which is extremely useful during the optimisation process. Additionally, the presented analytical solution requires less computational resources than, for example, FEM which, for microperiodic structures, must be based on a very refined mesh. Eventually, any adjustments in the microstructure of the considered beam are very convenient to implement when using averaged models. The whole structure is represented by the set of functions, such as *E*(*x*), *h*(*x*), ect. In order to alternate the considered beam one should change the form of those functions only, while the whole calculation algorithm remains exactly the same. As a result, it is easy to obtain a large number of calculation results of beams with various types of inhomogeneities using simple loops. On the other hand, the creation of multiple FEM models is usually a time-consuming process, which cannot always be completed with parametric modelling.

The formulas for the critical force parameters (10) and (12), presented in this paper, are derived for the most basic calculation example, in which the considered beam is characterised by simply supported boundary conditions. More complicated boundary conditions require the assumption of a more complex form of solution to the partial differential equation, cf. Equation (9). As a result, the derivation of the exact formulas for the critical force parameters may be very demanding for a complicated set of boundary conditions, contrary to the derivation of numerical solutions to the specific issue. Nevertheless, it can be noticed that the proposed averaged solutions are capable of covering such calculation cases. Let us notice, that both sets of the governing Equations (8) and (11) can be transformed into a single partial differential equation, whose form is similar to the classic partial differential equation of a homogeneous beam resting on the elastic foundation. Consequently, the applicable methods for the derivation of the critical force parameters in the case of the presented models are exactly the same as in the classic issues of structural mechanics.

## Figures and Tables

**Table 1:** The relative errors between TM and FEM models for Set I and k=1kPa.

		γ								
		0.1	0.2	0.3	0.4	0.5	0.6	0.7	0.8	0.9
ξ	0.1	7.55%	6.92%	6.33%	8.66%	16.55%	28.67%	38.82%	39.31%	23.18%
	0.2	6.72%	6.63%	5.62%	5.88%	9.19%	15.56%	21.75%	17.45%	5.27%
	0.3	5.04%	5.26%	4.40%	4.06%	5.46%	8.82%	11.66%	4.11%	2.99%
	0.4	3.52%	3.85%	3.22%	2.77%	3.31%	5.07%	1.89%	2.23%	1.74%
	0.5	2.33%	2.63%	2.22%	1.84%	2.00%	0.74%	1.10%	1.29%	1.00%
	0.6	1.45%	1.68%	0.70%	0.29%	0.30%	0.42%	0.61%	0.71%	0.56%
	0.7	0.20%	0.24%	0.21%	0.17%	0.17%	0.23%	0.31%	0.36%	0.29%
	0.8	0.11%	0.13%	0.12%	0.10%	0.09%	0.11%	0.14%	0.16%	0.14%
	0.9	0.05%	0.06%	0.06%	0.05%	0.05%	0.06%	0.06%	0.07%	0.06%
	1	0.04%	0.04%	0.04%	0.04%	0.04%	0.04%	0.04%	0.04%	0.04%

**Table 2:** The relative errors between ATM and FEM models for Set I and k=1kPa.

		γ								
		0.1	0.2	0.3	0.4	0.5	0.6	0.7	0.8	0.9
ξ	0.1	7.60%	7.00%	6.43%	8.77%	16.66%	28.75%	38.87%	39.32%	23.18%
	0.2	6.75%	6.68%	5.69%	5.96%	9.27%	15.63%	21.79%	17.45%	5.27%
	0.3	5.05%	5.29%	4.44%	4.12%	5.52%	8.87%	11.67%	4.11%	2.99%
	0.4	3.53%	3.87%	3.25%	2.80%	3.35%	5.10%	1.90%	2.23%	1.74%
	0.5	2.33%	2.64%	2.23%	1.86%	2.03%	0.75%	1.10%	1.29%	1.00%
	0.6	1.45%	1.68%	0.70%	0.29%	0.30%	0.43%	0.61%	0.71%	0.56%
	0.7	0.20%	0.24%	0.21%	0.18%	0.17%	0.23%	0.31%	0.36%	0.29%
	0.8	0.11%	0.13%	0.12%	0.10%	0.09%	0.11%	0.14%	0.16%	0.14%
	0.9	0.05%	0.06%	0.06%	0.05%	0.05%	0.06%	0.06%	0.07%	0.06%
	1	0.04%	0.04%	0.04%	0.04%	0.04%	0.04%	0.04%	0.04%	0.04%

**Table 3:** The relative errors between TM and FEM models for Set I and k=10kPa.

		γ								
		0.1	0.2	0.3	0.4	0.5	0.6	0.7	0.8	0.9
ξ	0.1	9.48%	8.57%	7.75%	10.49%	13.21%	22.01%	31.66%	35.62%	25.47%
	0.2	4.77%	4.75%	4.08%	4.37%	7.07%	12.45%	18.07%	19.43%	9.66%
	0.3	3.80%	4.01%	3.39%	3.18%	4.37%	7.24%	10.50%	9.32%	4.17%
	0.4	2.77%	3.05%	2.58%	2.24%	2.72%	4.24%	6.09%	3.15%	2.42%
	0.5	1.88%	2.15%	1.83%	1.52%	1.68%	2.45%	1.57%	1.81%	1.40%
	0.6	1.20%	1.40%	1.20%	0.98%	1.01%	0.61%	0.87%	1.00%	0.78%
	0.7	0.71%	0.83%	0.32%	0.26%	0.25%	0.33%	0.44%	0.51%	0.41%
	0.8	0.16%	0.19%	0.17%	0.15%	0.14%	0.17%	0.21%	0.23%	0.20%
	0.9	0.08%	0.09%	0.09%	0.08%	0.08%	0.08%	0.09%	0.10%	0.09%
	1	0.07%	0.07%	0.07%	0.07%	0.07%	0.07%	0.07%	0.07%	0.07%

**Table 4:** The relative errors between ATM and FEM models for Set I and k=10kPa.

		γ								
		0.1	0.2	0.3	0.4	0.5	0.6	0.7	0.8	0.9
ξ	0.1	9.67%	8.87%	8.14%	10.93%	13.47%	22.22%	31.78%	35.66%	25.48%
	0.2	4.82%	4.86%	4.23%	4.55%	7.26%	12.61%	18.17%	19.45%	9.67%
	0.3	3.83%	4.08%	3.48%	3.30%	4.50%	7.35%	10.57%	9.34%	4.17%
	0.4	2.78%	3.09%	2.63%	2.32%	2.81%	4.31%	6.14%	3.16%	2.43%
	0.5	1.89%	2.17%	1.86%	1.56%	1.73%	2.49%	1.58%	1.82%	1.40%
	0.6	1.21%	1.41%	1.22%	1.01%	1.04%	0.63%	0.88%	1.00%	0.78%
	0.7	0.71%	0.84%	0.32%	0.26%	0.26%	0.33%	0.45%	0.51%	0.41%
	0.8	0.16%	0.19%	0.17%	0.15%	0.14%	0.17%	0.21%	0.23%	0.20%
	0.9	0.08%	0.09%	0.09%	0.08%	0.08%	0.09%	0.09%	0.10%	0.09%
	1	0.07%	0.07%	0.07%	0.07%	0.07%	0.07%	0.07%	0.07%	0.07%

**Table 5:** The relative errors between TM and FEM models for Set I and k=100kPa.

		γ								
		0.1	0.2	0.3	0.4	0.5	0.6	0.7	0.8	0.9
ξ	0.1	8.88%	7.95%	7.25%	7.89%	15.20%	24.66%	30.87%	32.10%	23.82%
	0.2	6.01%	5.88%	5.01%	4.56%	6.24%	11.25%	16.65%	16.50%	9.89%
	0.3	3.29%	3.47%	2.94%	2.80%	3.93%	6.63%	9.48%	7.49%	5.58%
	0.4	2.45%	2.70%	2.29%	2.01%	2.48%	3.92%	3.73%	4.25%	3.24%
	0.5	1.70%	1.94%	1.65%	1.39%	1.55%	1.47%	2.13%	2.45%	1.87%
	0.6	1.10%	1.28%	0.87%	0.58%	0.60%	0.84%	1.18%	1.35%	1.05%
	0.7	0.42%	0.49%	0.44%	0.36%	0.35%	0.45%	0.61%	0.69%	0.56%
	0.8	0.23%	0.27%	0.25%	0.21%	0.20%	0.24%	0.29%	0.33%	0.28%
	0.9	0.13%	0.14%	0.14%	0.13%	0.12%	0.13%	0.14%	0.15%	0.14%
	1	0.10%	0.10%	0.10%	0.10%	0.10%	0.10%	0.10%	0.10%	0.10%

**Table 6:** The relative errors between ATM and FEM models for Set I and k=100kPa.

		γ								
		0.1	0.2	0.3	0.4	0.5	0.6	0.7	0.8	0.9
ξ	0.1	9.47%	8.87%	8.19%	8.92%	16.23%	25.23%	31.21%	32.19%	23.84%
	0.2	6.22%	6.29%	5.58%	5.06%	6.77%	11.70%	16.92%	16.57%	9.91%
	0.3	3.37%	3.64%	3.20%	3.13%	4.29%	6.94%	9.61%	7.55%	5.60%
	0.4	2.49%	2.80%	2.45%	2.22%	2.71%	4.12%	3.82%	4.29%	3.25%
	0.5	1.72%	2.00%	1.75%	1.52%	1.69%	1.55%	2.19%	2.47%	1.88%
	0.6	1.11%	1.32%	0.91%	0.62%	0.65%	0.88%	1.21%	1.36%	1.05%
	0.7	0.42%	0.50%	0.45%	0.38%	0.38%	0.48%	0.63%	0.70%	0.56%
	0.8	0.24%	0.27%	0.25%	0.22%	0.21%	0.25%	0.30%	0.33%	0.28%
	0.9	0.13%	0.14%	0.14%	0.13%	0.12%	0.13%	0.15%	0.15%	0.14%
	1	0.10%	0.10%	0.10%	0.10%	0.10%	0.10%	0.10%	0.10%	0.10%

**Table 7:** The relative errors between TM and FEM models for Set II.

		γ								
		0.1	0.2	0.3	0.4	0.5	0.6	0.7	0.8	0.9
*k*_2_[kPa]	0.1	0.07%	0.13%	0.10%	0.08%	0.07%	0.13%	0.13%	0.12%	0.11%
	0.5	0.07%	0.12%	0.10%	0.08%	0.07%	0.14%	0.13%	0.12%	0.11%
	1	0.06%	0.12%	0.10%	0.08%	0.07%	0.13%	0.12%	0.12%	0.11%
	5	0.15%	0.12%	0.09%	0.08%	0.13%	0.13%	0.12%	0.12%	0.11%
	10	0.13%	0.10%	0.09%	0.08%	0.14%	0.13%	0.12%	0.11%	0.11%
	20	0.10%	0.09%	0.08%	0.08%	0.14%	0.13%	0.12%	0.11%	0.11%
	40	0.07%	0.08%	0.14%	0.13%	0.13%	0.12%	0.11%	0.11%	0.11%
	60	0.13%	0.13%	0.12%	0.12%	0.12%	0.11%	0.11%	0.11%	0.11%
	80	0.11%	0.11%	0.11%	0.11%	0.11%	0.11%	0.11%	0.11%	0.10%

**Table 8:** The relative errors between ATM and FEM models for Set II.

		γ								
		0.1	0.2	0.3	0.4	0.5	0.6	0.7	0.8	0.9
*k*_2_[kPa]	0.1	0.07%	0.13%	0.10%	0.09%	0.07%	0.14%	0.13%	0.12%	0.11%
	0.5	0.07%	0.13%	0.10%	0.08%	0.07%	0.14%	0.13%	0.12%	0.11%
	1	0.06%	0.12%	0.10%	0.08%	0.08%	0.14%	0.13%	0.12%	0.11%
	5	0.15%	0.12%	0.10%	0.08%	0.14%	0.13%	0.13%	0.12%	0.11%
	10	0.13%	0.11%	0.09%	0.08%	0.14%	0.13%	0.12%	0.11%	0.11%
	20	0.11%	0.09%	0.08%	0.08%	0.14%	0.13%	0.12%	0.11%	0.11%
	40	0.07%	0.08%	0.14%	0.13%	0.13%	0.12%	0.11%	0.11%	0.11%
	60	0.13%	0.13%	0.12%	0.12%	0.12%	0.11%	0.11%	0.11%	0.11%
	80	0.11%	0.11%	0.11%	0.11%	0.11%	0.11%	0.11%	0.11%	0.10%

## Data Availability

Not applicable.
